# Perception of Health Care Practitioners of Government Designated COVID-19 Hospitals of Nepal towards the Management of COVID-19 Pandemic: A Descriptive Cross-sectional Study

**DOI:** 10.31729/jnma.7093

**Published:** 2022-01-31

**Authors:** Sagar Panthi, Amrit Raj Jaishi, Swotantra Gautam, Siddhartha Bhandari, Navin Bhatt, Lila Bahadur Basnet, Sanjib Kumar Sharma

**Affiliations:** 1 B. P. Koirala Institute of Health Sciences, Dharan, Nepal; 2 Institute of Medicine, Tribhuvan University Teaching Hospital, Kathmandu, Nepal; 3 Bayalpata Hospital, Achham, Nepal; 4 Ministry of Social Development, Lumbini Pradesh, Rupandehi, Nepal; 5 Department of Internal Medicine, B. P. Koirala Institute of Health Sciences, Dharan, Nepal

**Keywords:** *COVID-19*, *health care*, *pandemic*, *perception*

## Abstract

**Introduction:**

Amidst the chaos of COVID-19, health care practitioners are persistently providing services and experiencing many challenges. This study aimed to determine the perception of health care practitioners of government designated COVID-19 hospitals of Nepal towards the management of COVID-19 pandemic.

**Methods:**

A descriptive cross-sectional study was conducted among the frontline health care practitioners working in the government designated COVID-19 hospitals in Nepal from 21^st^ June, 2020 to 15^th^ August, 2020. Ethical approval was obtained from the Ethical Review Board of the Nepal Health Research Council (Reference number: 347/2020 P). A total of 252 health care practitioners (doctors, nurses, and paramedics) working at the forefront in the emergency ward, general wards, intensive care units, isolation centers, fever clinics, laboratory, quarantine centers, help desks, etc. in the designated hospitals who consented to participate were included in the study. Convenience sampling was used. The data was analyzed using Statistical Package for the Social Sciences version 16.0. Point estimate at 95% confidence interval was calculated along with frequency and proportion for binary data.

**Results:**

Only 41 (16.3%) (11.73-20.86 at 95% Confidence Interval) of the health care practitioners were found to have satisfactory perception towards the management of COVID-19 pandemic in Nepal.

**Conclusions:**

The satisfactory perception of the health care practitioners in our study towards the management of COVID-19 pandemic in Nepal is lower as compared to the other studies in Nepal and abroad.

## INTRODUCTION

While the health care practitioners (HCPs) are providing their services amidst COVID-19 pandemic, they are at constant risk of infecting themselves, their patients, their families, and their colleagues.^1^ Besides, in a low-resource setting like Nepal, due to the limited availability of protective equipment, additional challenges like overwhelming workload, widespread media coverage with unscientific information, lack of specific therapy, and feelings of inadequacy come forth.^1,2^

A study by Li Y, et al. suggests that the frontline HCPs go through peri-outbreak and post-outbreak psychological stress and sense a lack of self-security and the risk of transmission of infection to themselves and others.^3^ However, there is a dearth of literature regarding the perception of the HCPs during any infectious outbreak in Nepal.

Thus, with this study, we aimed to determine the perception of health care practitioners of government designated COVID-19 hospitals of Nepal towards the management of COVID-19 pandemic.

## METHODS

A descriptive cross-sectional study was conducted online among the frontline HCPs working in the government designated COVID-19 hospitals in Nepal from June 21, 2020 to August 15, 2020. Ethical approval was obtained from the Ethical Review Board of the Nepal Health Research Council (Reference number: 347/2020 P). 252 HCPs (doctors, nurses, and paramedics) working at the forefront in the emergency ward, general wards, intensive care units, isolation centers, fever clinics, laboratory, quarantine centers, help desks, etc. during the pandemic in the government designated COVID-19 hospitals who consented to participate were included in the study. Those who belonged to the specified hospitals but not present at work and the responses with incomplete data were excluded. Convenience sampling technique was used.

Sample size was calculated using the formula:

n = Z^2^ × (p × q) / e^2^

  = 1.96^2^ × 0.5 × 0.5 / (0.07)^2^

  = 196

Where,

n= required sample size
Z= 1.96 at 95% Confidence Interval (CI)
p= prevalence taken as 50% for maximum sample size
q= 1-p
e= margin of error, 7%

Considering non-respondent rate of 20%, total sample size becomes 235. However, our study included 252 HCPs.

With the physical means of transportation and communication under scrutiny due to the nationwide lockdown, and with the need to incorporate a large number of HCPs working in different areas of Nepal, this online-based study was deemed to be the most feasible one. The research questionnaire in Google form was distributed to the HCPs via email or social media platforms (Messenger, WhatsApp, etc.). The information and informed consent forms were included in the first page of the online questionnaire and e-consent was obtained by their approval to participate in the study.

Face and content validities of the questionnaire were ensured with judicial reference to the available literature and by seeking the opinion of the researchers and subject specialists respectively. Pretesting of the questionnaire was done among 20 HCPs who met the inclusion criteria; their responses were not included in the final dataset. To maintain confidentiality, only two researchers had access to the data repository stored on a password-protected computer.

The online questionnaire comprised of two main sections: perception of HCPs; and their sociodemographic characteristics. The perception section had 20 items with 3 on information dissemination, 7 on the impact of the pandemic, 4 on protective measures, and 6 on preparedness and management. The responses on perceptions were rated as Agree, Disagree, and Neutral using a 3-point Likert Scale. The perception was later categorized as "satisfactory" or "unsatisfactory". The sentences with affirmative intention and "agree" as the response were marked as satisfactory and a score of 1 was given. The other responses as "neutral" or "disagree" were given a score of 0. Cumulative percentage average of all the statements was done and those with an arbitrary percentage average of <50% were considered as "unsatisfactory perception" while those with > 50% as "satisfactory perception".^4,5^ The socio-demographic section included gender, age, address, designation, highest educational level, years of medical practice, working hours per shift, an experience of working in an outbreak, practice setup, workplace distribution of COVID-19 positive and suspected cases, and deaths in the hospital.

The responses were obtained in the form of a spreadsheet and then checked to avoid any duplications or technical errors. The dataset was created with necessary coding using Statistical Package for the Social Sciences (SPSS) version 16.0 after confirming the completeness of the data. Sociodemographic characteristics were analyzed using descriptive statistics. Point estimate at 95% Confidence Interval was calculated and along with frequencies, percentages, mean, and standard deviations.

## RESULTS

On an overall evaluation of the perception of HCPs, only 41 (16.3%) (11.73-20.86 at 95% Confidence Interval) of the participants were found to have satisfactory perception towards information dissemination, protective measures, and preparedness & management of the COVID-19 pandemic while the remaining 211 (83.7%) of the HCPs were found to have unsatisfactory perception. Only 89 (35.3%) felt that the information on COVID-19 provided to the HCPs was complete and updated. Similarly, 44 (17.5%) were found to be satisfied with the media providing updated information. Three fourth 183 (72.6%) felt that this pandemic revealed the staff inadequacy at their hospital while only 50 (19.8%) believed that their hospital is prepared enough to combat the crisis. Two-thirds of the participants 173 (68.7%) considered hand hygiene and PPE as effective in preventing transmission to the HCPs. Only one-third 93 (36.9%) felt that they were given sufficient training on PPE use. Only 55 (21.8%) were satisfied with the higher health care authorities on handling the outbreak and containing the infection. About one-third 85 (33.7%) found the diagnostic test services at their facility accessible and reliable. Only 112 (44.4%) of the HCPs agreed that their hospital provided them with clear guidelines on managing and handling COVID-19 cases (Table 1).

**Table 1:** Perception of health care practitioners on the COVID-19 pandemic.

Perception	Agree n (%)	Neutral n (%)	Disagree n (%)
**Perception on information dissemination**			
Information on COVID-19 provided to the health care providers is complete, well timed and updated.	89 (35.3)^*^	100 (39.7)	63 (25.0)
Information on COVID-19 provided to the general public is complete, well timed and updated.	65 (25.8)^*^	123 (48.8)	64 (25.4)
Media is providing correct and complete information on COVID-19 and not creating a state of infodemics.	44 (17.5)^*^	138 (54.8)	70 (27.8)
**Perception on impact of pandemic**			
I was afraid of the risk of transmission of the disease to the family members due to the high risk of my exposure.	213 (84.5)	11 (4.4)	28 (11.1)^*^
Pandemic resulted in increased workload on me.	149 (59.1)	57 (22.6)	46 (18.3)^*^
I was afraid that the people would avoid me or my family members at my place of residence.	170 (67.5)	37 (14.7)	45 (17.9)^*^
I feel very stressed and anxious due to my increased workload and the thought of being infected.	115 (45.6)	76 (30.2)	61 (24.2)^*^
This period of crisis would result in inadequate staffs at my hospital to handle the volume of work.	183 (72.6)	41(16.3)	28 (11.1)^*^
I feel my hospital is well prepared to combat this period of crisis.	50 (19.8)^*^	64 (54.8)	64 (25.4)
Besides me, I feel my choice of work has resulted in a state of fear and anxiety among my family members as well.	192 (76.2)	27 (10.7)	33(13.1)^*^
**Perception On Protective Measures**			
Hand Hygiene and PPE are effective and self-sufficient in preventing most of the infection transmission risk to the health workers.	173 (68.7)^*^	38 (15.1)	41 (16.3)
We were given sufficient training and guidelines on PPE use (how to wear, when to wear, how to dispose of, etc.)	93(36.9)^*^	123 (48.8)	36 (14.3)
There was no disparity in distribution of PPE to health care providers working at different levels of qualification.	90 (35.7)^*^	126 (50.0)	36 (14.3)
The PPE were comfortable to work with and didn't hamper my usual performance.	30 (12.0)^*^	174 (69.0)	48 (19.0)
**Perception On Preparedness and Management**			
I feel lockdown was the best measure to tackle the transmission of infection in our setup.	132 (52.4)^*^	60 (23.8)	60 (23.8)
I think the government and the health care authorities did a fine job with handling the outbreak and help in containing the spread of infection.	55 (21.8%)^*^	133 (52.8)	64 (25.4)
The diagnostic test service was accessible and reliable at my facility.	85 (33.7)^*^	112 (44.4)	55 (21.8)
My hospital has provided us with a definitive protocol to handle the cases of corona infection with a clear guideline on which cases to be reported, tested, or admitted.	112 (44.4)^*^	96 (38.1)	44 (17.5)
It is always good that the health care professional who is attending the patient also collect the samples to avoid extra use of PPE.	137 (54.4)^*^	78 (31.0)	37 (14.7)
I think prescribing anti-viral drugs which were used in Influenza or 37 (14.7) previous corona outbreaks will also be effective against COVID-19 infection (SARS-COV-2).	87 (34.5)	128 (50.8)^*^

* a= satisfactory perception of the health care providers working during COVID-19 pandemic

Socio-demographic details revealed the median age of the respondents as 27 years (range: 20 to 44 years). Maximum participants were from Province 1 69 (27.4%) and Bagmati 65 (25.8%) while Karnali 15 (6%) had the least. Most of the involved HCPs were doctors 154 (61.1) followed by nurses 64 (25.4%) and paramedics 34 (13.5%). About half of the participants 121 (48%) had an experience of clinical practice of one to five years while only a quarter 62 (24.6%) had that of more than 5 years. Only 31 (12.3%) of the respondents had worked in an infectious outbreak or a pandemic before. Workplace distribution showed that most of the respondents worked in the general wards 146 (57.9%) followed by emergency 113 (44.8%) and isolation ward 88 (34.9%). About 205 (81.3%) of the HCPs had encountered positive cases at their facility while 208 (82.5%) had encountered suspected cases with 77 (30.5%) encountering COVID-19 related deaths (Table 2).

**Table 2:** Socio-demographic details.

Characteristics		n (%)
Age in years	Median age	27 years
Gender	Male	138 (54.8)
(M:F=1.21:1)	Female	114 (45.2)
Address	Province 1	69 (27.4)
	Province 2	18 (7.1)
	Bagmati Pradesh	65 (25.8)
	Gandaki Pradesh	26 (10.3)
	Lumbini Pradesh	31 (12.3)
	Karnali Pradesh	15 (6.0)
	Sudurpashchim Pradesh	28 (11.1)
Education Level	Graduate	200(79.4)
	Post-Graduate	52 (20.6)
Designation	Doctor	154 (61.1)
	Nurse	64 (25.4)
	Paramedic	34 (13.5)
Years of medical practice	≤ 1 year	69 (27.4)
	1-5 years	121 (48.0)
	> 5 years	62 (24.6)
Working hour per shift	≤ 8 hours	141 (55.9)
	9-16 hours	74 (29.4)
	>16 hours	37 (14.7)
Past experience working in an infectious outbreak	Yes	31 (12.3)
	No	221 (87.7)
Practice Setup	Rural	65 (25.8)
	Semi-urban	50 (19.8)
	Urban/Metro-city	137 (54.4)
Workplace Distribution	Emergency	113 (44.8)
	ICU	68 (26.9)
	General Wards	146 (57.9)
	Fever Clinics	80 (31.7)
	Help desk	29 (11.5)
	Sample Collection center	33 (13.1)
	Laboratory	24 (9.5)
	Isolation	88 (34.9)
	Quarantine center	43 (17.6)
	Others	10 (4.0)
Positive COVID-19 cases in hospital	Yes	205 (81.3)
	No	47 (18.7)
Suspected cases in isolation	Yes	208 (82.5)
	No	17.5)
COVID-19 Deaths in the hospital	Yes	77 (30.5)
	No	175 (69.5)

## DISCUSSION

COVID-19 pandemic has affected every country, person, and profession. With the health care system being confronted with unprecedented challenges, the frontline HCPs face new obstacles. Sadly, very little has been addressed about these issues in Nepal. This study is one of the very few which studied the perception of the HCPs working in government designated COVID-19 hospitals of Nepal towards the management of COVID-19 pandemic.

In our study, only 13.6% of the HCPs were found to have satisfactory perception with the information dissemination, protective measures, preparedness, and management of the COVID-19 pandemic. This is similar to another study of Nepal which reports a very low satisfaction among the healthcare workers (36.4%) towards the government's response to the pandemic.^6^ Likewise, general practitioners and practice assistants in the Netherlands during the 2009 H1N1 Influenza pandemic also expressed lower satisfaction towards government-provided patient information and the guidelines on PPE use.^7^ Contrary to this finding is a study done in the United Arab Emirates where high satisfaction (78%) was exhibited by the HCPs towards transmission prevention strategies in COVID-19 pandemic.^8^ A similar response was obtained from the Nepalese public where around 71.4% of the study population was satisfied with government response to the early phase of the pandemic.^9^ This perception difference between HCPs and the general public is understandable. Direct involvement of HCPs in rendering health care while risking their own lives can significantly affect their perception according to the theoretical models on job conditions.^10,11^

Theoretical models on job conditions suggest that the individuals working in environments with excess demands, shortage of resources, and lack of control over their work may develop mental health issues depression and burnouts.^10,11^ In our study, a majority of the HCPs were afraid of transmitting the disease to their family members and had a stressful workload. A recent review on the mental impact suggested that the HCPs exposed to infectious patients had 1.7 times greater odds of developing psychological distress and Post Traumatic Stress Disorders (PTSD).^12^ Such stress-related anxiety and fear have also been reported in many studies done abroad during this pandemic1,2,^13-18^ and also in previous pandemics like Ebola.^3^ To cope up with these issues, studies suggest the need for an active psychological and social support system, regular monitoring of staff wellbeing, and provision of evidence-based treatments.^17,19,20^

A study done in the United States during COVID-19 pandemic suggests increased infection vulnerability among the HCPs because of changing treatment guidelines, management protocols, and potential PPE shortages.^1^ In our study too, HCPs felt the staff inadequacy at their hospitals during the pandemic, confronted the lack of sufficient training on PPE use and only a few were satisfied with the higher health care authorities on handling the outbreak and a lack of the provision of clear guidelines by the hospitals on managing and handling COVID-19 cases. These responses concur with studies done among HCPs in Nepal and abroad.^6,7,21,22^

Few studies done abroad during COVID-19 and Ebola pandemics identified excessive workloads, incorrect use of PPE, lack of proper training on infectious outbreaks, limited provision of emergency command system, and losing control over the situation at work as the main reasons responsible for moral distress among the HCPs.^3,16,23^ This distress may be attributed to the lack of similar experience which is in accordance with our study, as only 12.3% of the respondents were found to possess experience of working in an infectious outbreak. This is quite supported by a study conducted during the 2009 H1N1 Influenza A pandemic where HCPs with a clinical experience of >11 years were found to be more compliant on using protective gears.^24^ Thus, provision of adequate equipment and proper training for medical teams is needed to boost their morale during pandemics.^3^

A review on the responses of the general practitioners to public health crises highlighted constraints especially in terms of information access, access to PPE, outbreak training, and emotional support, and advocated the need for improving pandemic preparedness and encouraging willingness to work during the crisis.^25,26^ Likewise, there is a need to systematically lower factors like job stress, time pressure, and limited organizational support which led to professional burnout.^27^ A UK based study suggested the provision for clear and consistent management guidelines; regular testing of the healthcare providers, and adequate administration of PPEs.^18^ The 2009 H1N1 Influenza pandemic offered us valuable insights over providing information to the frontline HCPs and encouraging policymakers in planning and preparing for future pandemics.^28^

To prepare ourselves to combat pandemics in the future, it is important to address the perceptions of the HCPs. Policymakers and the authorities in the health care sectors need to consider the issues of security in terms of protective equipment, incentives, and provision of a safe place for isolation and treatment if they or their family members get infected. Though the findings cannot be generalized as this study has covered a very small proportion of HCPs involved, it can still give us an insight into their perceptions and guide the authorities to frame a suitable environment for the HCPs to work efficiently.

## CONCLUSIONS

In our study, the proportion of the HCPs with satisfactory perception towards the clinical and administrative management of COVID-19 pandemic in Nepal is lower as compared to the studies in Nepal and abroad. This underlines the central dogma of the mental status of a health care practitioner during the pandemic; with training inadequacy leading to work dissatisfaction which inevitably has the potential to impact patient care and further clinical outcomes.
